# Direct impact of COVID-19 vaccination in Chile: averted cases, hospitalizations, ICU admissions, and deaths

**DOI:** 10.1186/s12879-024-09304-1

**Published:** 2024-05-03

**Authors:** Antoine Brault, Andrew Hart, Paula Uribe, Jorge Prado, Jaime San Martín, Alejandro Maass, Mauricio Canals

**Affiliations:** 1 Centro de Modelamiento Matemático, Universidad de Chile and CNRS IRL2807, Santiago, Chile; 2 Mathematical Modelling of Infectious Diseases Unit, Institut Pasteur, Université Paris Cité, CNRS UMR 2000, Paris, F-75015 France; 3 Departamento de Ingeniería Matemática, Facultad de Cs. Físicas y Matemáticas, Universidad de Chile, Santiago, Chile; 4 Millennium Institute Center for Genome Regulation, Santiago, Chile; 5 Departamento de Medicina (O) y Programa de Salud Ambiental, Facultad de Medicina, Escuela de Salud Pública, Universidad de Chile, Santiago, Chile

**Keywords:** SARS-CoV-2, COVID-19, Vaccine, Averted events, Cases averted, Hospitalizations averted, ICU admissions averted, Deaths averted

## Abstract

**Background:**

Chile rapidly implemented an extensive COVID-19 vaccination campaign, deploying a diversity of vaccines with a strategy that prioritized the elderly and individuals with comorbidities. This study aims to assess the direct impact of vaccination on the number of COVID-19 related cases, hospital admissions, ICU admissions and deaths averted during the first year and a half of the campaign.

**Methods:**

Via Chile’s transparency law, we obtained access to weekly event counts categorized by vaccination status and age. Integrating this data with publicly available census and vaccination coverage information, we conducted a comparative analysis of weekly incidence rates between vaccinated and unvaccinated groups from December 20, 2020 to July 2, 2022 to estimate the direct impact of vaccination in terms of the number of cases, hospitalizations, ICU admissions and deaths averted, using an approach that avoids the need to explicitly specify the effectiveness of each vaccine deployed.

**Results:**

We estimated that, from December 20, 2020 to July 2, 2022 the vaccination campaign directly prevented 1,030,648 (95% Confidence Interval: 1,016,975-1,044,321) cases, 268,784 (95% CI: 264,524-273,045) hospitalizations, 85,830 (95% CI: 83,466-88,194) ICU admissions and 75,968 (95% CI: 73,909-78,028) deaths related to COVID-19 among individuals aged 16 years and older. This corresponds to a reduction of 26% of cases, 66% of hospital admissions, 70% of ICU admissions and 67% of deaths compared to a scenario without vaccination. Individuals 55 years old or older represented 67% of hospitalizations, 73% of ICU admissions and 89% of deaths related to COVID-19 prevented.

**Conclusions:**

This study highlights the role of Chile's vaccination campaign in reducing COVID-19 disease burden, with the most substantial reductions observed in severe outcomes.

**Supplementary Information:**

The online version contains supplementary material available at 10.1186/s12879-024-09304-1.

## Background

As of November 2023, the COVID-19 pandemic has resulted in over 772 million cases and 6.98 million deaths worldwide [1]. In Chile, more than 5.3 million cases and 62,000 deaths have been reported [2]. At the beginning of the pandemic, the country implemented non-pharmaceutical interventions, such as school and university closures, lockdowns, cordons sanitaires, and curfews [3]. Measures such as social distancing, hand sanitization and mask-wearing were also instituted, the latter remaining in effect until the end of September, 2022 [4]. Furthermore, in response, Chile underwent one of the most rapid and extensive vaccination campaigns in the world [5]. This began at the end of December 2020, with the immunization of healthcare workers, followed by a massive campaign to vaccinate the general population prioritized by age and comorbidity, which started in February 2021. Chile was also among the first countries to initiate a booster-vaccination campaign, which started in early August 2021. Originally aimed at individuals who had received the full CoronaVac vaccination, the booster-vaccination campaign was later expanded to include all fully vaccinated individuals. Adherence by the population to the vaccination program was remarkably high; by the end of 2021, 84% of the country's 19.5 million inhabitants had received a full course of vaccination while 56% had received a booster dose. Vaccination coverage has continued to increase during 2022 and 2023; as at October 2, 2023, 94.3% of the population aged 18 years and older has completed a primary course of vaccination (one or two doses, according to the protocol for administering each vaccine), 93.1% have received a booster dose, and 82.5% had received a fourth dose [6].

**Fig. 1:**
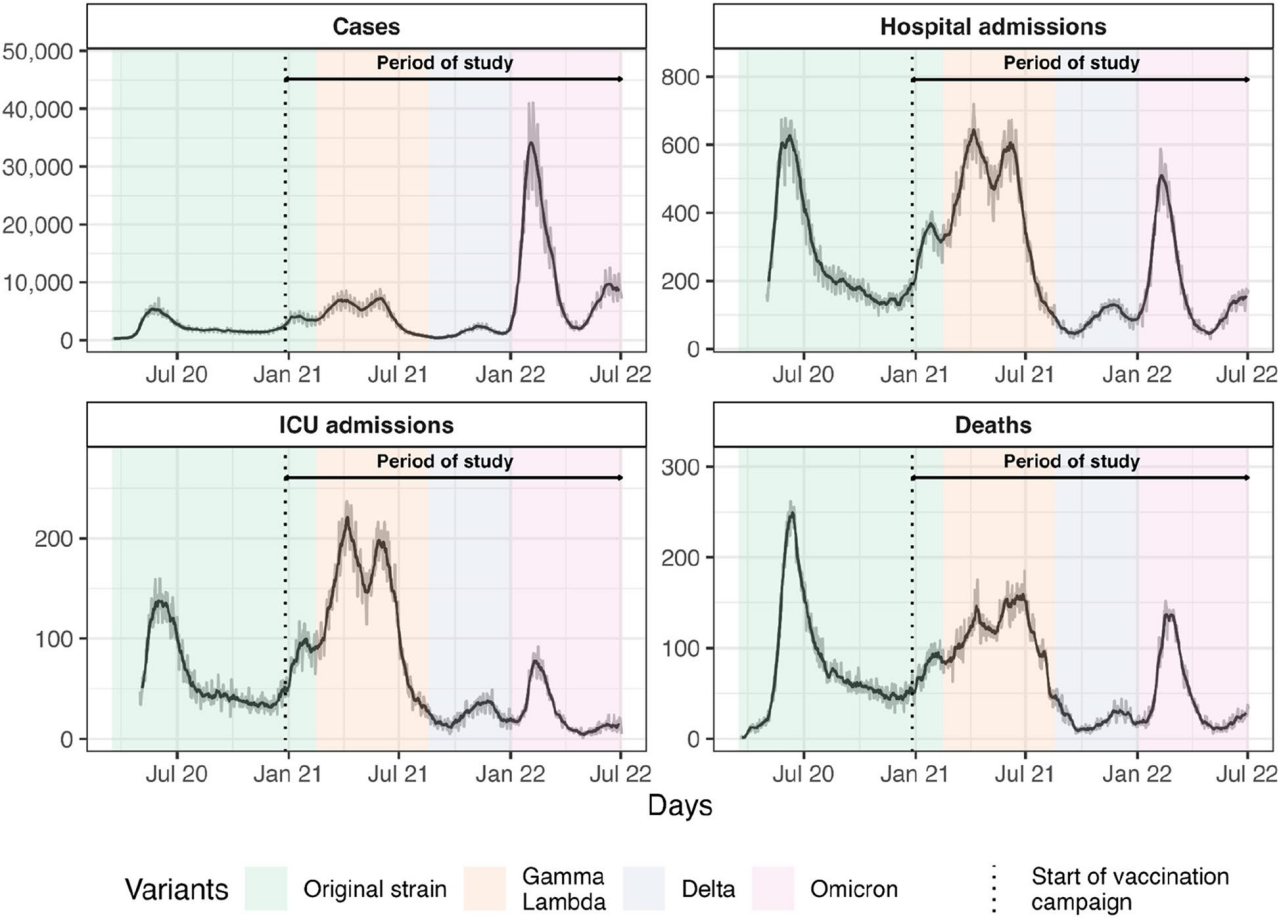
Temporal trends of COVID-19 related cases, hospitalizations, ICU admissions and deaths in Chile. The gray lines represent daily data, while the black lines show the 7-day rolling mean. The horizontal arrow indicates the period of study (from the beginning of the vaccination campaign on December 20, 2020, to July 2, 2022). The vertical dotted line marks the start of the vaccination campaign. The colors represent the time intervals during which each variant was prevalent. Note that healthcare facilities only started recording data sometime after the pandemic began

Chile experienced successive waves of COVID-19 cases (Fig. 1). The first of these began in March 2020 with the original strain of COVID-19, followed by a important wave due to the Gamma and Lambda variants between February and August of 2021. Subsequently, a wave driven by the Delta variant emerged in mid-August 2021. In January 2022, the Omicron variant started to spread in Chile and this remains the dominant variant today [7].

Assessments of the impact of SARS-CoV-2 vaccination campaigns have been conducted predominantly in high-income nations, where mRNA vaccines have been extensively employed [8–12]. CoronaVac, an inactivated virus vaccine, has been widely used in Chile. As at July 2, 2022, it accounted for 43.9% of administered doses. Other vaccines, including BNT162b2, mRNA-1273, ChAdOx1, Ad5-nCoV and Ad26.COV2.S, were also administered. To our knowledge, there are few studies measuring the direct impact of vaccination in a country where the CoronaVac vaccine has been widely deployed [13].

Earlier research in Chile has evaluated the effectiveness of the CoronaVac vaccine during the initial phases of the vaccination campaign [14], as well as the antibody response elicited by CoronaVac compared to individuals who received the BNT162b2 vaccine [15]. Despite the remarkable scope of Chile's vaccination campaign, its overall impact remains unknown. One international study has estimated the number of lives saved during the first year of global vaccination with a particular focus on Chile [16], but it drew upon limited data from Chile and made substantial assumptions about vaccine effectiveness, which is challenging to determine in Chile due to the number of vaccines deployed and differences in how much their effectiveness have been studied.

Quantifying averted outcomes attributable to the vaccination campaign nationally would be valuable, not only for helping to assess the benefits of vaccination but also for informing the design of mass vaccination campaigns in response to future pandemics. In this study, we take advantage of comprehensive data on COVID-related outcomes, categorized by age group and vaccine-administration schedules, to accurately estimate the direct impact of the first year and a half (December 20, 2020 - July 2, 2022) of vaccination in terms of cases, hospitalizations, intensive-care-unit (ICU) admissions and deaths averted in Chile in individuals aged 16 years and older.

## Methods

### Model selection

Various approaches have been suggested for estimating the number of averted outcomes prevented by vaccination programs [12, 17–20]. We chose to implement the method introduced in Haas et al. [6] which uses the difference between incidence rates between vaccinated and unvaccinated groups to derive the number of outcomes averted. The method was compatible with the available data on incidence by vaccination status and circumvented the need for vaccine effectiveness estimates that could not be accurately quantified for all the vaccines used in Chile.

### Data and information sources

The vaccination program started at the end of December 2020, and the study period extended until July 2, 2022, focusing on individuals aged 16 years and older. Six different vaccines were administered during the period of study. The most widely used vaccine was CoronaVac, developed by Sinovac Biotech, accounting for 43.9% of administered doses. This was followed by BNT162b2 (brand name Comirnaty), developed by Pfizer and BioNTech, which made up 42.2% of the doses administered. Next, the Moderna-developed vaccine mRNA-1273 (brand name Spikevax) covered 7.5% of administered doses. ChAdOx1 nCoV-19 (brand name Vaxzevria), developed by AstraZeneca, constituted 5.4% of doses, while Ad5-nCoV (brand name Convidecia) developed by CanSino Biologics and Ad26.COV2.S developed by Janssen Pharmaceutica accounted for the remaining 1% of all doses administered.

Data was procured from three sources. The first was data obtained by means of a freedom-of-information (known as the Law of Transparency in Chile) request to the Transparency Unit of the Subsecretary of Public Health. This data included the numbers of COVID-19-related cases, hospitalizations, ICU admissions and deaths by epidemiological week (with Sunday as the first day of the week), categorized by age group (0-15, 16-24, 25-34, 35-44, 45-54, 55-64, 65-74, 75-84 and >=85) and number of vaccine doses received (taking values in the range from 0 to 4, with 0 denoting unvaccinated).

In Chile, a case of COVID-19 is defined as an individual who has received a positive result for SARS-CoV-2 from a PCR or antigenic test. Hospitalization or ICU admission related to COVID-19 is declared by medical staff of the receiving healthcare center when the reason for the patient visit is due to COVID-19. When determining whether a death is related to COVID-19, medical authorities make a judgment based on a range of factors such as the individual's medical history, symptoms and available test results.

Next, we generated the total number of individuals who had received 1, 2, 3 or 4 doses of vaccine by age for each week based on aggregated data from the Chilean national immunization registry (detailed in Supplementary Material 1).

We derived the third data set from Chilean population statistics broken down by age, which were obtained from the last national census conducted in 2017 [21]. We separately aggregated the 2021 and 2022 population projections for individual ages to obtain estimates for the same age groups as those used in the other data sets. We then obtained the final population estimates used in this study by adding two-thirds of the 2021 estimate and one-third of the 2022 estimate in each age range. The ratio (2: 1) selected for combining the estimates was based on the study period covering the whole of 2021 but only half of 2022.

### Statistical analysis

We estimated the number of cases, hospitalizations, ICU admissions and deaths averted among individuals aged 16 years and older who received 1, 2, 3 and 4 vaccine doses by adapting the method presented in Haas et al. [8]. We computed specific daily incidence rates for each age group (16-24, 25-34, 35-44, 45-54, 55-64, 65-74, 75-84, and >=85), considering individuals who had received exactly 0, 1, 2, 3 or 4 vaccine doses. Next, for each epidemiological week of the analyzed period, we calculated the differences in incidence rate between the group with 0 doses and the groups with 1, 2, 3 and 4 doses. Then the number of events averted is given by (Supplementary Material 1 for details):

$${\sum }_{t = Epi.\ week\ starting\ Dec.\ 20,\ 2020}^{Epi.\ week\ ending\ July\ 2,\ 2022}{\sum }_{a = 1}^{8}{\sum }_{v =1}^{3}{{N}_{a}^{susc}\left(t\right)p}_{a,v}\left(t\right)\left({ I}_{a,0}\left(t\right)-{I}_{a,v}\left(t\right)\right),$$

Where

- ${N}_{a}^{susc}(t)$ is the number of susceptible individuals in epidemiological week $t$, in age range $a$, that is, the number of individuals in age group $a$ less the number of cases that occurred in that age group up to week $t$.
- ${p}_{a,v}(t)$ is the proportion of individuals vaccinated with exactly $v$ doses in age group $a$ in epidemiological week $t$.
- ${I}_{a,v}(t)$ is the incidence rate (number of events divided by the number of individuals) in epidemiological week $t$, in age group $a$, in the group that received exactly $v$ vaccine doses.

According to the above formula, we computed the number of averted events in age group $a,$ for vaccine dose number $v$, in epidemiological week $t$ as ${{N}_{a}^{susc}(t)p}_{a,v}(t)({ I}_{a,0}(t) - {I}_{a,v}(t))$.

Confidence intervals for the number of outcomes averted were calculated using the normal approximation of differences in proportions, assuming that the incidence rates are independent. The analysis was conducted using version 4.3.1 of the R statistical software.

### Sensitivity analysis

We performed a first sensitivity analysis in which we assumed that individuals who have previously been infected have the same susceptibility to reinfection and severe outcomes as those who have never been infected. Additional details regarding this assumption can be found in Section 1.2 of the Supplementary Material 1 (Counterfactual Scenario A). This involves substituting ${N}_{a}^{susc}(t)$ by the total population of the given age group $a$.

Given the uncertainty regarding the Chilean population, we conducted a second sensitivity analysis in which the age pyramid corresponds to that of 2021, rather than the baseline scenario, which aggregates two-thirds of 2021 and one-third of 2022.

## Results

### Covid-19 epidemic and vaccination campaign

**Fig. 2:**
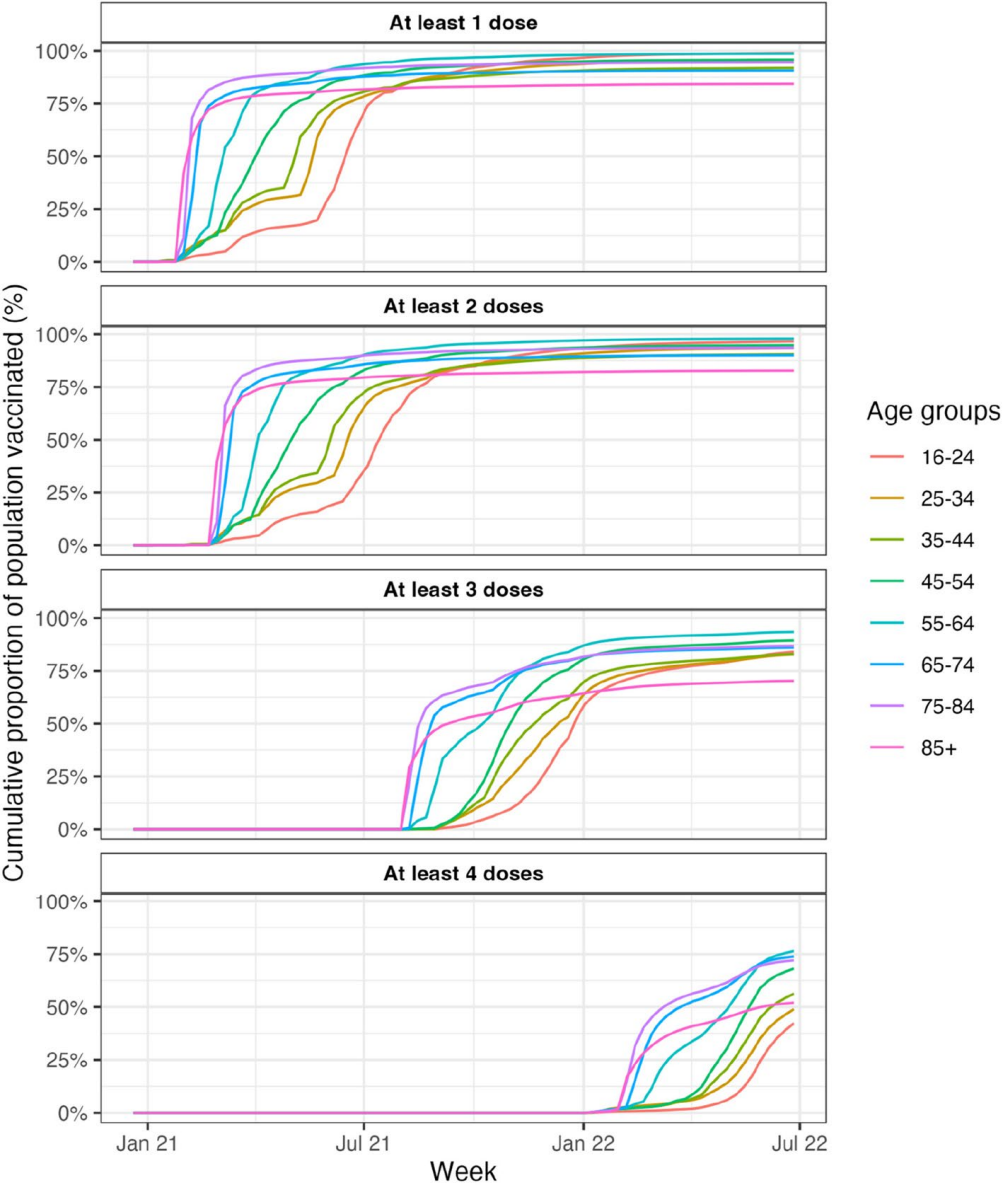
Cumulative proportion of the population vaccinated by age and each week in Chile

At the end of the study period (July 2, 2022), 14,981,425 (95%) out of 15,740,549 Chileans aged 16 years or older had received at least one dose, 14,761,706 (94%) had received at least two doses, 13,510,471 (86%) had received at least three doses and 9,393,909 (60%) had received four doses. Vaccine doses were initially administered to healthcare workers starting on December 20, 2020, and the mass vaccination campaign began in early February 2021, prioritizing the elderly and individuals with comorbidities (Fig. 2). At the end of August 2021, the first-booster (third-dose) vaccination campaign began, which continued to prioritize older individuals and vulnerable groups. The second-booster (fourth-dose) campaign commenced at the beginning of January, 2022, coinciding with the start of the Omicron wave in Chile. The second analysis modifies the assumptions by substituting the age pyramid from the 2021 census for the two-to-one combination of 2021 and 2022 census data used in the baseline scenario.

### Number of events directly averted by vaccination

**Fig. 3:**
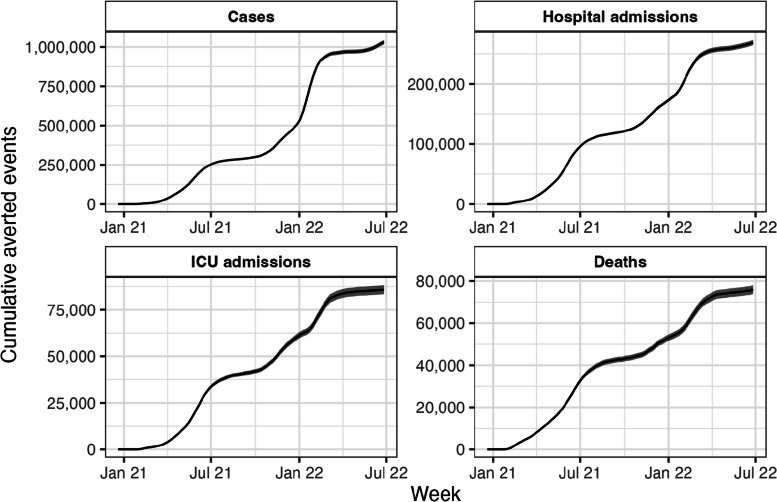
Cumulative averted events in individuals 16 years of age and older in Chile. Each plot represents respectively the cumulative number of cases, hospital admissions, ICU admissions and deaths related to COVID-19 averted between December 20, 2020, and July 2, 2022, due to vaccination against COVID-19

We estimated that 1,030,648 cases (95% Confidence Interval: 1,016,975-1,044,321), 268,784 (95% CI: 264,524**-**273,045) hospitalizations, 85,830 (95% CI: 83,466-88,194) ICU admissions, and 75,968 (95% CI: 73,909-78,028) deaths related to COVID-19 were directly averted by vaccination among individuals aged 16 years or older between December 20, 2020 and July 2, 2022. It represents a reduction of 26% of cases, 66% of hospitalizations, 70% of ICU admissions and 67% of deaths with respect to a scenario without vaccination. Figure 3 shows a three-stage time series of the averted events. The first increase in the cumulative number of averted events between March and July 2021 corresponds to the administration of the second dose during the mass vaccination campaign and a wave of new cases due to the Lambda and Gamma variants. A second rise starting from October 2021 coincides with the booster-dose vaccination campaign and the Delta-variant wave while the third increase starting from January 2022 corresponds to the wave caused by the Omicron-variant.

Most preventions of severe outcomes were observed in individuals vaccinated with two doses: 125,472 (95% CI: 123,453-127,491) hospitalizations, 43,113 (95% CI: 41,955-44,272) ICU admissions and 40,036 (95% CI: 38,806-41,267) deaths related to COVID-19 (Figure S1). Individuals 55 years old or older represented 30% of the Chilean population over 16 years old, but accounted for 42% of cases, 67% of hospitalizations, 73% of ICU admissions and 89% of deaths related to COVID-19 prevented (Table 1).

**Table 1:** Estimated number of COVID-19 related outcomes averted by age and number of doses received

**Age**	**Cases**	**Hospitalizations**	**ICU admissions**	**Deaths**
**Averted outcomes in individuals vaccinated with 1 dose**				
16-24	10487 (10034 to 10940)	644 (574 to 714)	81 (55 to 107)	21 (9 to 33)
25-34	9372 (8843 to 9901)	1738 (1620 to 1857)	508 (444 to 571)	78 (54 to 102)
35-44	-723 (-1172 to -273)	1401 (1264 to 1538)	511 (433 to 590)	124 (89 to 158)
45-54	6625 (6195 to 7056)	2547 (2382 to 2711)	940 (842 to 1038)	405 (352 to 458)
55-64	11565 (11085 to 12045)	4110 (3884 to 4336)	1560 (1422 to 1698)	942 (844 to 1039)
65-74	-31 (-342 to 280)	1055 (868 to 1242)	396 (285 to 507)	805 (681 to 929)
75-84	2067 (1791 to 2342)	1724 (1530 to 1919)	379 (288 to 471)	1690 (1513 to 1867)
>=85	141 (21 to 261)	184 (104 to 264)	9 (-13 to 30)	449 (357 to 541)
**Total 1 dose**	**39503 (38367 to 40638)**	**13403 (12962 to 13844)**	**4384 (4138 to 4630)**	**4513 (4250 to 4777)**
**Averted outcomes in individuals vaccinated with 2 doses**				
16-24	125159 (122424 to 127893)	4745 (4305 to 5184)	566 (425 to 708)	122 (57 to 187)
25-34	78857 (77181 to 80534)	7784 (7443 to 8126)	2134 (1964 to 2303)	396 (316 to 475)
35-44	20329 (19282 to 21377)	9640 (9294 to 9987)	3147 (2957 to 3338)	821 (713 to 929)
45-54	65632 (64148 to 67117)	23326 (22610 to 24043)	8432 (8006 to 8857)	3788 (3483 to 4092)
55-64	119905 (117577 to 122232)	50461 (49030 to 51891)	19688 (18809 to 20567)	13355 (12573 to 14136)
65-74	8468 (7505 to 9430)	14894 (14220 to 15568)	6172 (5754 to 6589)	7361 (6899 to 7824)
75-84	16697 (15665 to 17728)	13031 (12273 to 13788)	2916 (2563 to 3268)	11651 (10951 to 12351)
>=85	591 (224 to 958)	1591 (1346 to 1836)	59 (-3 to 121)	2543 (2253 to 2833)
**Total 2 doses **	**435638 (431041 to 440235)**	**125472 (123453 to 127491)**	**43113 (41955 to 44272)**	**40036 (38806 to 41267)**
**Averted outcomes in individuals vaccinated with 3 doses**				
16-24	259680 (252940 to 266421)	9612 (8540 to 10684)	646 (360 to 932)	219 (51 to 387)
25-34	78806 (75071 to 82540)	7031 (6465 to 7596)	992 (796 to 1188)	160 (77 to 243)
35-44	-72970 (-75080 to -70860)	4152 (3795 to 4508)	1068 (900 to 1236)	315 (220 to 410)
45-54	30690 (27209 to 34171)	13511 (12632 to 14390)	3923 (3468 to 4377)	1889 (1559 to 2218)
55-64	278524 (270325 to 286723)	61236 (58157 to 64316)	22811 (20960 to 24663)	11945 (10603 to 13287)
65-74	-42672 (-43787 to -41556)	6666 (6143 to 7189)	2764 (2460 to 3067)	2460 (2180 to 2741)
75-84	1672 (427 to 2918)	9962 (9224 to 10701)	2412 (2066 to 2758)	5626 (5121 to 6132)
>=85	-4503 (-4850 to -4156)	616 (431 to 802)	19 (-28 to 65)	1211 (1035 to 1386)
**Total 3 doses**	**529228 (517141 to 541314)**	**112787 (109222 to 116351)**	**34634 (32635 to 36633)**	**23824 (22301 to 25347)**
**Averted outcomes in individuals vaccinated with 4 doses**				
16-24	29621 (27734 to 31509)	947 (658 to 1236)	19 (-1 to 39)	3 (-1 to 7)
25-34	-8056 (-8985 to -7128)	717 (586 to 848)	51 (19 to 83)	11 (-1 to 22)
35-44	-25087 (-25712 to -24461)	282 (200 to 363)	42 (15 to 70)	41 (14 to 67)
45-54	-9485 (-10594 to -8376)	897 (699 to 1094)	170 (90 to 249)	150 (81 to 219)
55-64	55758 (52363 to 59154)	7541 (6652 to 8431)	1956 (1584 to 2327)	2019 (1582 to 2455)
65-74	-15683 (-16231 to -15135)	1632 (1415 to 1849)	611 (501 to 722)	971 (835 to 1107)
75-84	970 (245 to 1696)	4631 (4206 to 5056)	848 (673 to 1023)	3381 (3046 to 3716)
>=85	-1760 (-1966 to -1553)	476 (372 to 580)	2 (-22 to 26)	1020 (914 to 1126)
**Total 4 doses**	**26279 (21985 to 30573)**	**17123 (16039 to 18208)**	**3699 (3263 to 4134)**	**7594 (7013 to 8176)**
**Total of all doses**	**1030648 (1016975 to 1044321)**	**268784 (264524 to 273045)**	**85830 (83466 to 88194)**	**75968 (73909 to 78028)**

### Sensitivity analysis 1: entire population remains susceptible to infection

The first sensitivity analysis assumed a scenario in which the entire population remains susceptible to reinfection and severe outcomes over time (see counterfactual scenario A in Section 1.2 of the Supplementary Material 1 for more details). Under this assumption, we estimated that a total of 1,124,060 (95% CI: 1,108,627-1,139,492) cases, 290,142 (95% CI: 285,460-294,824) hospitalizations, 92,065 (95% CI: 89,485-94,645) ICU admissions, and 80,979 (95% CI: 78,745-83,214) deaths related to COVID-19 were prevented (Table S1). These estimates represent an increase of 9% for cases, 8% for hospitalizations, 7% for ICU admissions and 7% for deaths averted relative to the baseline scenario where infections detected over time were excluded. Thus, the results are fairly robust with respect to the population assumed to be susceptible.

### Sensitivity analysis 2: 2021 population census

The second analysis modifies the assumptions by substituting the age pyramid from the 2021 census for the two-to-one combination of 2021 and 2022 census data used in the baseline scenario. This adjustment yields a slightly different count of averted events spanning the study period (Figure S2), but the difference becomes much more pronounced with the onset of the Omicron wave in January 2022.

At the end of the study period, we estimate that 1,281,719 (95% CI: 1,264,750-1,298,689) cases were averted, 329,941 (95 % CI: 324,111-335,770) hospitalizations, 106,553 (95% CI: 103,236-109,870) ICU admissions and 95,571 (95% CI: 92,753-98,389) deaths were directly averted by vaccination. This connotes an important increase of 24% in cases, 23% in hospitalizations, 24% in ICU admissions and 26% in deaths averted with respect to the baseline scenario.

The substantial difference is due to the continuing reduction in the unvaccinated population as the vaccination campaign progresses. Consequently, the impact of the choice of census estimate used to compute this population becomes increasingly important over time.

## Discussion

We employed a method that directly assesses the impact of the vaccination program by comparing the weekly incidence of various outcomes between vaccinated and unvaccinated individuals over time, similar to the approaches taken in Israel and Brazil [8, 13]. The results of our study demonstrate the substantial impact of Chile's vaccination campaign in reducing the burden of COVID-19 in terms of cases, hospitalizations, ICU admissions and deaths during the first year and a half of the campaign. We estimate that vaccination efforts prevented 26% of cases, 66% of hospitalizations, 70% of ICU admissions, and 67% of deaths among individuals aged 16 years or older during the study period. Notably, the most significant reductions were observed for the severe outcomes of hospitalization, ICU admission and death, shedding light on the critical role vaccination has played in mitigating the severity of COVID-19.

Our findings also revealed that the complete vaccination with 2 doses had an important impact on preventing COVID-19-related severe outcomes, emphasizing the importance of completing the full course of vaccination. Moreover, the first-booster (third-dose) campaign contributed to further reductions in cases and severe outcomes during the Delta variant wave, which supports the role of booster doses in prolonging vaccine effectiveness.

It is important to note that individuals over 55 years old, who represented 30% of the Chilean population of at least 16 years of age, accounted for the majority of prevented hospitalizations, ICU admissions and deaths. This observation highlights the effectiveness of Chile's age-based prioritization strategy in targeting the most vulnerable populations and reducing the overall burden on healthcare systems.

Our estimations of averted outcomes are robust to hypothesis on the population susceptible (sensitivity analysis 1), but are sensitive to the census data on which we base our calculation of the size of the unvaccinated population (sensitivity analysis 2).

Watson et al. [16] conducted an international study to evaluate the number of deaths averted by COVID-19 vaccination in 185 countries and territories, with a focus on Chile. They estimated that between December 8, 2020, and December 8, 2021, 108,100 (94,170 - 121,800) and 146,100 (137,200 - 163,700) deaths were averted in Chile based on COVID-19-related deaths and excess mortality, respectively. These estimates are higher than our estimates of deaths averted, which cover a longer period. This is due firstly to the fact that we focus solely on the direct effect of vaccination whereas Watson et al. take into account the effect of reduced transmission among vaccinated individuals. However, they make assumptions about vaccine effectiveness based on studies of other vaccines and variants [22], as such studies were not available for Coronavac. If Coronavac is less effective at preventing transmission than mRNA vaccines, this could lead to an overestimation of the number of averted deaths.

To the best of our knowledge, the only publication addressing events averted in a country that made extensive use of the CoronaVac vaccine is Santos et al. [13], who estimated that 82% of COVID-19-related deaths were averted in Brazil between January 17, 2021, and January 31, 2022. This is higher than our estimate of 67%. The discrepancy may be attributed to differences in the study period, types of vaccines used, as well as the influence of geography and regional policies for managing the epidemic within the two countries.

In the Additional file 1, we showed the connection between our approach and a conventional method employed to assess vaccination impact through vaccine effectiveness [15–18]. However, we were unable to use this method due to unavailability of comprehensive data on vaccine effectiveness for the different types of vaccines, number of doses, against different variants for all vaccines deployed in Chile.

Our study is not without limitations. First of all, we have not taken into account the indirect impact of vaccination due to reduced transmission among vaccinated individuals. However, this would require precise knowledge of vaccine effectiveness against transmission and infection against each variant for each vaccine. To our knowledge, such data are not available in the literature. Although our methodology based on comparing incidence rates between vaccinated and unvaccinated populations allows us to avoid making assumptions about vaccine effectiveness, it is still subject to potential biases. It was not possible to account for confounding variables such as co-morbidities, socioeconomic strata and testing rates, etc. Furthermore, we did not consider any difference in detection rates between vaccinated and unvaccinated subjects, which may skew the estimate of averted cases, but probably has less impact on estimates of severe outcomes averted. Finally, we should note that our method can lead to negative estimates in the number of cases averted (Table 1). The diminished effectiveness of initial vaccine generations against the Omicron strain, coupled with the waning effectiveness of vaccines over time [23], could partially account for this phenomenon. Additionally, vaccinated individuals may exhibit behaviors that put them at a higher risk of infection compared to their unvaccinated counterparts.

## Conclusions

This study shows Chile's vaccination campaign sharply reduced the burden of COVID-19. Our findings underscore the importance of widespread vaccination, particularly among vulnerable populations, in controlling the pandemic and alleviating the strain on healthcare systems.

### Supplementary Information

**Supplementary Material 1.** 

## Data Availability

Data and code are available on request from Antoine Brault at abrault@pasteur.fr.

## Abbreviations

COVID-19: Coronavirus disease 2019
SARS-CoV-2: Severe acute respiratory syndrome coronavirus 2
ICU: Intensive care unit
mRNA: Messenger ribonucleic acid
$N_a^{susc}(t)$: Number of susceptible individuals in epidemiological week $t$, in age group $a$
${p}_{a,v}(t)$: Proportion of individuals vaccinated with exactly $v$ doses in age group $a$ in epidemiological week $t$
$I_{a,v}(t)$: Incidence rate (number of events divided by the number of individuals) in epidemiological week $t$, in age group $a$, in the group that received exactly $v$ vaccine doses
