# Supplementary material for: Direct impact of COVID-19 vaccination in Chile: averted cases, hospitalizations, ICU admissions, and deaths
Source: BMC Infect Dis. 2024 May 3;24:467. doi: 10.1186/s12879-024-09304-1 (PMC11067253; doi:10.1186/s12879-024-09304-1)
Supplement: Supplementary file 1 — Supplementary Material 1. [file 12879_2024_9304_MOESM1_ESM.pdf]

Supplementary Material 1

Direct impact of COVID-19 vaccination in Chile: averted cases, hospitalizations, ICU admissions, and deaths

Antoine Brault, Andrew Hart, Paula Uribe, Jorge Prado, Jaime San Martín, Alejandro Maass, Mauricio Canals

1. Estimation of averted outcomes by vaccination....................1
  1.1 Notation....................2
  1.2 Derivation of the formula of averted outcomes....................2
  1.3 Link with vaccine effectiveness....................5
2. Table of averted events by dose and age for counterfactual scenario A (sensitivity analysis 1)......... 7
3. Averted per vaccination schedule....................9
4. Averted events in sensitivity analysis 2....................10
5. Data for computing vaccination coverage....................11
References....................12

1. Estimation of averted outcomes by vaccination

In the literature we found two main methods to estimate the direct impact of vaccination in terms of outcomes averted. One introduced in Lévy-Bruhl [18] is based on vaccine effectiveness. This method, primarily designed to quantify the impact of the BCG vaccination, has been widely employed in studying the effects of influenza vaccination [15–17] and more recently to estimate the impact of vaccination against COVID-19 [10]. The other, introduced by Haas et al. [6] was derived from the difference in incidence rates between vaccinated and unvaccinated groups. Here, we examine the relationship between these two methodologies.

1.1 Notation

We use the following notation:

- m_v is the maximum number of vaccine doses considered
- m_a is the number of age groups
- $N_{a,v}(t)$ is the number of individuals who have received exactly v vaccine doses in age category a at time t ($N_{a,0}(t)$ corresponds to the number of individuals who have received 0 doses, that is, are unvaccinated)
- $N_a(t)$ is the number of individuals in age category a at time t
- $p_{a,v}(t) = N_{a,v}(t)/N_a(t)$ is the proportion of the population vaccinated with exactly v doses in age category a at time t
- $C_{a,v}(t)$ is the number of cases among those vaccinated with exactly v doses in age category a that occur at time t
- $I_{a,v}(t) = C_{a,v}(t)/N_a(t)$ is the incidence rate in the group of individuals vaccinated with exactly v doses in age category a at time t ($I_{a,0}(t)$ is the incidence rate for unvaccinated individuals in age category a at time t)

1.2 Derivation of the formula of averted outcomes

We begin by providing a justification for the formula used to calculate averted outcomes in our study. Although we specifically derive the formula for the number of cases averted, the same formula is applicable to other outcomes as well. The derivation of the formula is based on the following assumption: in **counterfactual scenario A (CFA)** where the population is unvaccinated, the number of cases $C_a^{CFA}(t) = I_{a,0}(t)N_a(t)$ would have occurred in age group a at time t. Consequently, the number of cases averted in age group a at time t is given by

$$C_a^{Averted}(t) = C_a^{CFA}(t) - \sum_{v=0}^{m_v} C_{a,v}(t)$$

$$= I_{a,0}(t)N_a(t) - \sum_{v=0}^{m_v} I_{a,v}(t)N_{a,v}(t)$$

$$= \sum_{v=0}^{m_v} (I_{a,0}(t)N_{a,v}(t) - I_{a,v}(t)N_{a,v}(t))$$

$$= \sum_{v=1}^{m_v} (I_{a,0}(t) - I_{a,v}(t))N_{a,v}(t)$$

$$= \sum_{v=1}^{m_v} p_{a,v}(t)N_a(t)(I_{a,0}(t) - I_{a,v}(t)).$$

The number of events averted by dose v in age group a at time t is then

$$C_{a,v}^{Averted}(t) = p_{a,v}(t)N_a(t)(I_{a,0}(t) - I_{a,v}(t)).$$

Moreover, it follows that the total number of cases averted over a period ranging from t = 1 to t = T is obtained by summing this expression over all age categories a and times t:

$$\sum_{t=1}^{T} \sum_{a=1}^{m_a} \sum_{v=1}^{m_v} p_{a,v}(t)N_a(t)(I_{a,0}(t) - I_{a,v}(t)) . \qquad (1)$$

In Haas et al. [5] it was assumed that events do not occur in individuals exhibiting evidence of prior infection. This corresponds to another scenario without vaccination, say **counterfactual scenario B (CFB),** where $C_a^{CFB}(t) = I_{a,0}^{susc}(t)N_a^{susc}(t)$, where $N_a^{susc}(t)$ represents the

susceptible population in age group a at time t, calculated as the total population in age group a less the cumulative case count up to time t in this age group and $I_{a,0}^{susc}(t)$ denotes the incidence rate of events in the unvaccinated susceptible population. If we further assume that vaccination coverage is consistent across both the susceptible and general populations, then the total number of events averted is

$$\sum_{t=1}^{T} \sum_{a=1}^{m_a} \sum_{v=0}^{m_v} p_{a,v}(t) N_a^{susc} (I_{a,0}^{susc}(t) \quad - I_{a,v}^{susc}(t)) . \qquad (2)$$

It is often infeasible to compute $I_{a,v}^{susc}(t) = C_{a,v}^{susc}(t)/N_{a,v}^{susc}(t)$ from aggregated data as this requires follow-up of individuals across vaccination categories. However, according to our hypothesis of non reinfection $C_{a,v}^{susc}(t) = C_{a,v}(t)$ and assuming that the number of infections is low compared to the population within each age group, we can use the approximation $I_{a,v}^{susc}(t) \simeq I_{a,v}(t)$. Hence the total number of events averted is given by

$$\sum_{t=1}^{T} \sum_{a=1}^{m_a} \sum_{v=1}^{m_v} p_{a,v}(t) N_a^{susc}(t) (I_{a,0}(t) \quad - I_{a,v}(t)) . \qquad (3)$$

In this study, we employed formula (3) in our baseline scenario, and conducted a sensitivity analysis using formula (1). It should be noted that formula (3) always yields a lower (more conservative) estimate of averted events in comparison to formula (1). However, it is not clear to us whether Haas et al. [5] utilized Equation (2) or Equation (3) in estimating the burden of events averted in their study.

1.3 Link with vaccine effectiveness

In Lévy-Bruhl [1], Bonmarin et al. [2], Foppa et al. [3] and Machado et al. [4], the calculation of the number of events (such as infections, hospitalizations and deaths) averted by influenza vaccination was performed using a formula that adjusts an estimate for the number of excess events in the absence of vaccination by vaccine coverage and vaccine effectiveness:

$$C_a^{Averted}(t) = C_a(t)\frac{\sum_{v=1}^{m_v} p_{a,v}(t)VE_{a,v}(t)}{1-\sum_{v=1}^{m_v} p_{a,v}(t)VE_{a,v}(t)}. \quad (4)$$

Here, we examine the relationship between Equations (4) and (1). More precisely, we shall show that if the vaccine effectiveness in age group a at time t is defined as $VE_{a,v}(t) = 1 - I_{a,v}(t)/I_{a,0}(t)$, then the two equations are equivalent.

Consider the ratio of the number of events that occur when some of the population is vaccinated to the number of events that occur under counterfactual scenario A in which nobody is vaccinated. This is

$$\frac{C_a(t)}{C_a^{CFA}(t)} = \sum_{v=0}^{m_v} \frac{C_{a,v}(t)}{I_{a,0}(t)N_a(t)}$$

$$= \sum_{v=0}^{m_v} \frac{C_{a,v}(t)\, N_{a,0}(t)}{C_{a,0}(t)N_a(t)}$$

$$= \frac{N_{a,0}(t)}{N_a(t)} + \sum_{v=1}^{m_v} \frac{C_{a,v}(t)\, N_{a,0}(t)\, N_{a,v}(t)}{C_{a,0}(t)N_a(t)\, N_{a,v}(t)}$$

$$= p_{a,0}(t) + \sum_{v=1}^{m_v} p_{a,v}(t)\frac{I_{a,v}(t)}{I_{a,0}(t)}$$

$$\frac{C_a(t)}{C_a^{CFA}(t)} = \sum_{v=0}^{m_v} \frac{C_{a,v}(t)}{I_{a,0}(t)N_a(t)}$$

$$= 1- \sum_{v=1}^{m_v} p_{a,v}(t) + \sum_{v=1}^{m_v} p_{a,v}(t)\frac{I_{a,v}(t)}{I_{a,0}(t)}$$

$$= 1- \sum_{v=1}^{m_v} p_{a,v}(t)(1 - \frac{I_{a,v}(t)}{I_{a,0}(t)})$$

$$= 1- \sum_{v=1}^{m_v} p_{a,v}(t)VE_{a,v}(t) \qquad (5)$$

Then, the number of cases averted is given by

$$C_a^{Averted}(t) = C_a^{CFA}(t) - C_a(t)$$

$$= C_a(t)(\frac{C_a^{CFA}(t)}{C_a(t)} - 1)$$

$$= C_a(t)\frac{\sum_{v=1}^{m_v} p_{a,v}(t)VE_{a,v}(t)}{1- \sum_{v=1}^{m_v} p_{a,v}(t)VE_{a,v}(t)},$$

where we use Equation (5) to relate $C_a^{CFA}(t)$ and $C_a(t)$. Note that if we assume counterfactual scenario B, then formula (4) still holds true but with $VE_{a,v}(t) = 1 - I_{a,v}^{susc}(t)/I_{a,0}^{susc}(t)$. In this scenario, $VE_{a,v}(t)$ is more akin to the measures vaccine effectiveness computed in observational studies where individuals

who have been infected previously are excluded from consideration in the study.

2. Table of averted events by dose and age for counterfactual scenario A (sensitivity analysis 1)

Age	Cases	Hospital admissions	ICU admissions	Deaths
**Averted outcomes in individuals vaccinated with 1 dose**				
16-24	11894 (11393 to 12395)	710 (632 to 787)	88 (60 to 116)	23 (10 to 36)
25-34	10102 (9530 to 10674)	1862 (1735 to 1989)	543 (475 to 611)	84 (58 to 110)
35-44	-1005 (-1480 to -530)	1468 (1324 to 1612)	537 (454 to 620)	130 (93 to 167)
45-54	6868 (6418 to 7317)	2658 (2487 to 2830)	983 (881 to 1085)	423 (367 to 479)
55-64	12165 (11669 to 12662)	4276 (4043 to 4509)	1624 (1482 to 1767)	979 (877 to 1080)
65-74	-63 (-379 to 253)	1059 (870 to 1249)	399 (287 to 512)	810 (684 to 936)
75-84	2100 (1821 to 2379)	1751 (1553 to 1948)	385 (293 to 478)	1712 (1533 to 1891)
>=85	142 (20 to 264)	186 (105 to 268)	8 (-14 to 30)	453 (360 to 547)
**Total 1 dose**	**42204 (41001 to 43406)**	**13969 (13513 to 14426)**	**4568 (4314 to 4822)**	**4614 (4345 to 4883)**
**Averted outcomes in individuals vaccinated with 2 doses**				
16-24	139315 (136260 to 142371)	5275 (4784 to 5766)	619 (463 to 776)	136 (63 to 209)
25-34	86503 (84637 to 88369)	8459 (8083 to 8834)	2304 (2119 to 2488)	428 (341 to 515)
35-44	20554 (19422 to 21686)	10229 (9858 to 10600)	3340 (3137 to 3544)	875 (760 to 990)
45-54	69400 (67817 to 70982)	24690 (23929 to 25450)	8921 (8468 to 9373)	4015 (3692 to 4339)
55-64	127286 (124823 to 129749)	53174 (51666 to 54683)	20740 (19813 to 21666)	14081 (13256 to 14905)
65-74	8488 (7495 to 9481)	15325 (14630 to 16021)	6361 (5929 to 6792)	7583 (7105 to 8061)
75-84	17189 (16124 to 18253)	13454 (12672 to 14237)	3014 (2650 to 3378)	12027 (11304 to 12750)

>=85	557 (176 to 938)	1631 (1377 to 1884)	60 (-5 to 125)	2618 (2318 to 2919)
**Total 2 doses**	**469292 (464299 to 474286)**	**132237 (130107 to 134366)**	**45359 (44140 to 46577)**	**41763 (40478 to 43049)**
**Averted outcomes in individuals vaccinated with 3 doses**				
16-24	294626 (286845 to 302408)	11016 (9774 to 12258)	745 (410 to 1079)	253 (58 to 448)
25-34	86139 (81799 to 90479)	8113 (7454 to 8773)	1132 (905 to 1358)	186 (88 to 284)
35-44	-86744 (-89136 to -84352)	4620 (4218 to 5022)	1187 (998 to 1375)	356 (248 to 464)
45-54	31603 (27722 to 35484)	14923 (13946 to 15899)	4306 (3804 to 4808)	2114 (1744 to 2485)
55-64	306360 (297310 to 315409)	67224 (63833 to 70615)	25030 (22990 to 27069)	13187 (11702 to 14673)
65-74	-46433 (-47627 to -45239)	7057 (6500 to 7614)	2937 (2613 to 3260)	2622 (2323 to 2922)
75-84	1569 (241 to 2897)	10600 (9815 to 11386)	2559 (2192 to 2925)	6017 (5478 to 6556)
>=85	-4887 (-5260 to -4515)	646 (447 to 844)	18 (-31 to 68)	1292 (1104 to 1480)
**Total 3 doses**	**582233 (568617 to 595849)**	**124199 (120255 to 128143)**	**37912 (35710 to 40115)**	**26027 (24349 to 27706)**
**Averted outcomes in individuals vaccinated with 4 doses**				
16-24	36111 (33796 to 38427)	1154 (799 to 1509)	23 (-1 to 47)	4 (-1 to 8)
25-34	-10233 (-11413 to -9053)	893 (726 to 1060)	63 (22 to 103)	13 (-1 to 27)
35-44	-30662 (-31432 to -29892)	344 (243 to 444)	51 (17 to 85)	50 (17 to 83)
45-54	-11392 (-12725 to -10058)	1066 (828 to 1304)	201 (105 to 296)	177 (95 to 260)
55-64	65323 (61303 to 69344)	8796 (7746 to 9846)	2263 (1828 to 2699)	2357 (1842 to 2871)
65-74	-17766 (-18384 to -17148)	1820 (1576 to 2064)	681 (557 to 805)	1085 (932 to 1238)
75-84	958 (146 to 1770)	5143 (4668 to 5618)	943 (748 to 1138)	3757 (3384 to 4131)
>=85	-2008 (-2239 to -1777)	520 (404 to 636)	1 (-25 to 28)	1132 (1013 to 1250)
**Total 4 doses**	**30331 (25195 to 35468)**	**19736 (18462 to 21010)**	**4226 (3720 to 4732)**	**8574 (7904 to 9245)**
**Total of all doses**	**1124060 (1108627 to 1139492)**	**290142 (285460 to 294824)**	**92065 (89485 to 94645)**	**80979 (78745 to 83214)**

Table S1. Estimated number of COVID-19-related outcomes averted for counterfactual scenario A (sensitivity analysis 1).

3. Averted outcomes per vaccination schedule

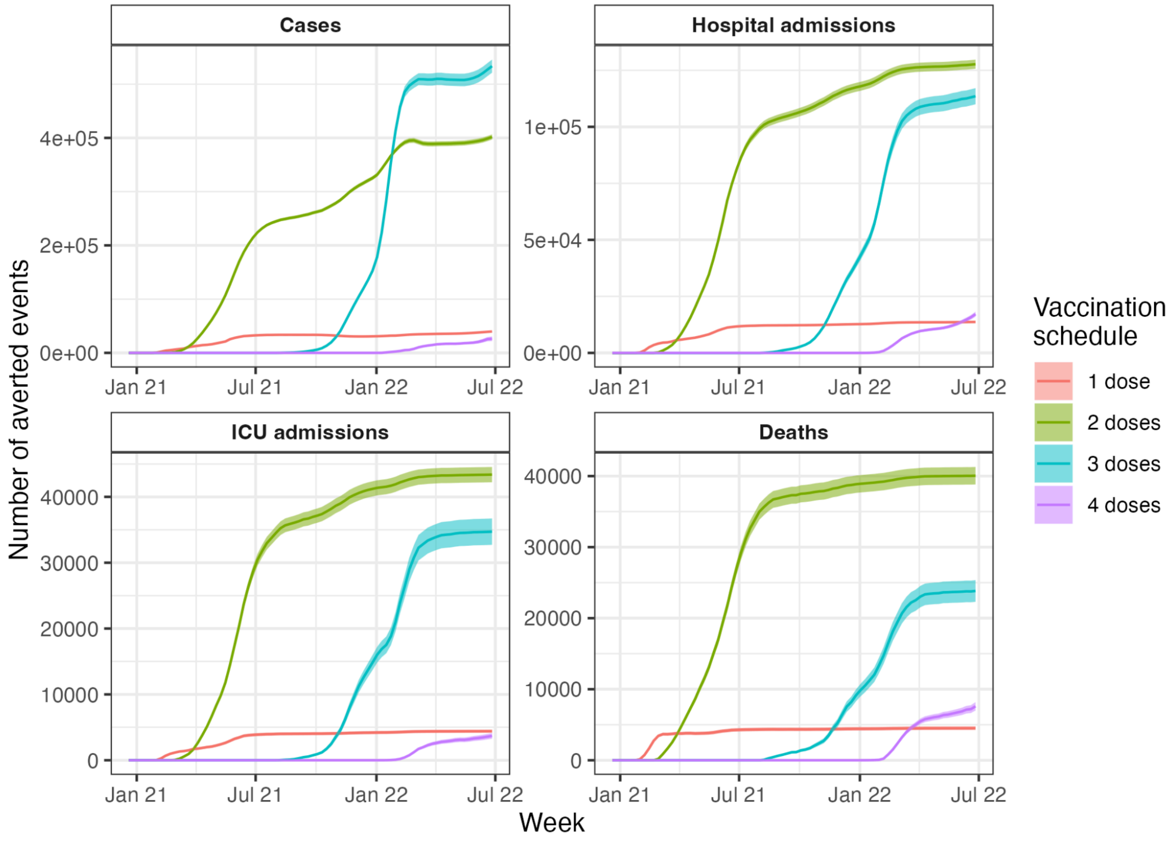

Figure S1: Cumulative COVID-19 related outcomes (cases, hospital admissions, ICU admissions, deaths) averted in individuals 16 years of age and older in Chile per vaccination schedule. In red, individuals were vaccinated with 1 dose, in green with 2 doses, in blue with 3 doses and in purple with 4 doses.

4. Averted outcomes in sensitivity analysis 2

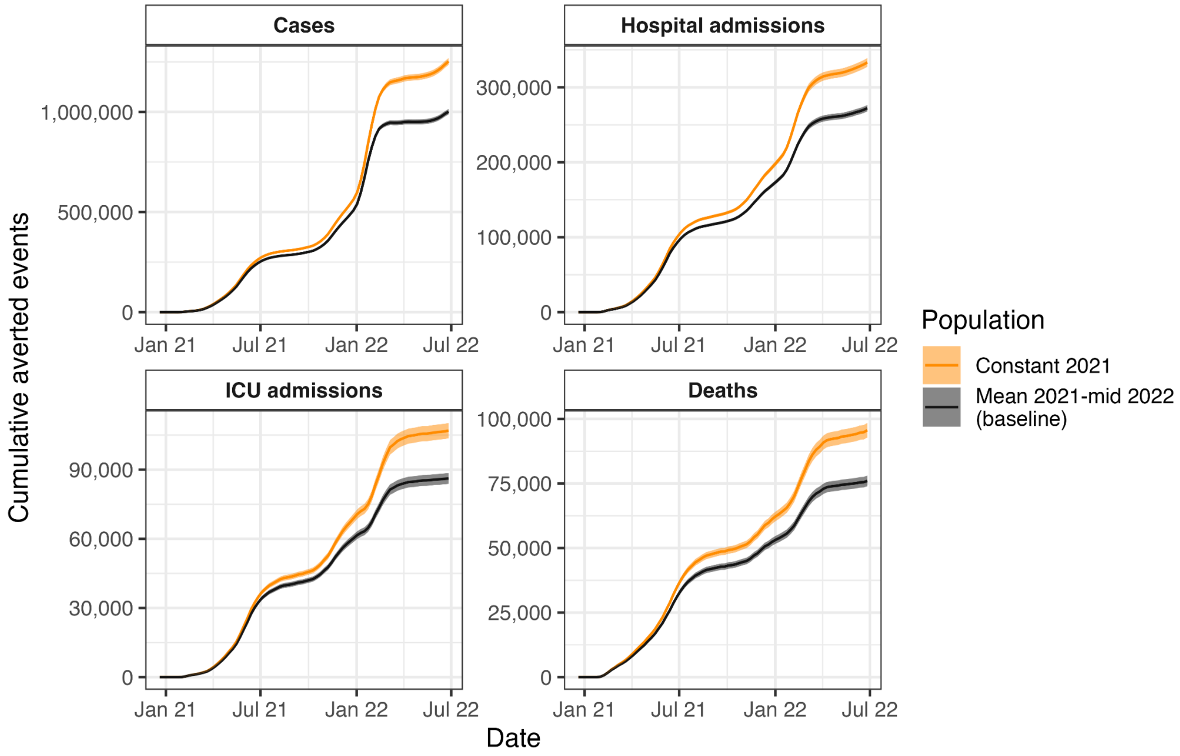

Figure S2: Cumulative COVID-19 related outcomes (cases, hospital admissions, ICU admissions, deaths) averted in individuals 16 years of age and older in Chile. In orange, sensitivity analysis scenario 2 using the age pyramid from the 2021 census; in black, the baseline scenario considering the age pyramid derived from two-thirds of the 2021 census and one-third of the 2022 census estimates.

5. Data for computing vaccination coverage

We made use of aggregated data from the Chilean national immunization registry (RNI) to compute the vaccination coverage by age group. The data are available in the GitHub repository [6] operated by the Ministry of Science, Technology, Knowledge and Innovation. We made use of five files from Product 78 (in the output/producto78 directory) which were submitted to the repository in the commit with hash D305B2161 dated January 6th, 2023. For reasons unknown to the authors, the repository is no longer online.These five files, one for each kind of dose recorded—first dose, second dose, third dose, fourth dose and single dose (for vaccines like Ad5-nCoV and Ad26.COV2.S that only require a single dose to confer protection)—provide the number of vaccine doses administered on each date to individuals of each age. However, this data set consists of counts of injections administered, not counts of individuals vaccinated. To make it compatible with the incidence data provided by the Ministry of Health, it was necessary to transform it into the number of individuals with one, two, three and four doses of vaccine. To do this exactly, it would be necessary to have individual COVID-19 vaccination histories. Lacking such, we computed counts by employing the following procedure. First, for each age group, dose administered and date, we computed the cumulative total for the dose up to and including the date. These cumulative sums are the only quantities used in the following description. To calculate the number of people who had received their first dose, we subtracted the number of second doses administered from the number of first doses administered. Similarly, the number of individuals having exactly two doses was obtained as the number of second doses administered less the number of third doses administered. Then, the number of individuals with three doses was computed as the difference between the number of third doses administered and fourth doses administered. Given that no more than four doses were administered during the study period, the number of fourth doses administered corresponds to the number of individuals who had received

precisely four doses. Finally, the number of individuals administered a single-dose vaccine (such as Ad5-nCoV and Ad26.COV2.S) was added to the number of people having exactly two doses, both corresponding to the status of being fully vaccinated. Consequently, “third dose” and “fourth dose” refer respectively to the first booster dose and second booster dose received by an individual.

References

1. Lévy-Bruhl D. Estimation de l’impact épidémiologique de différentes options de vaccination BCG en France. Revue d’Épidémiologie et de Santé Publique. 2005;53:501–8.

2. Bonmarin I, Belchior E, Lévy-Bruhl D. Impact of influenza vaccination on mortality in the French elderly population during the 2000-2009 period. Vaccine. 2015;33:1099–101.

3. Foppa IM, Cheng PY, Reynolds SB, Shay DK, Carias C, Bresee JS, Kim IK, Gambhir M, Fry AM. Deaths averted by influenza vaccination in the U.S. during the seasons 2005/06 through 2013/14. Vaccine. 2015;33:3003–9.

4. Machado A, Mazagatos C, Dijkstra F, Kislaya I, Gherasim A, McDonald SA, et al. Impact of influenza vaccination programmes among the elderly population on primary care, Portugal, Spain and the Netherlands: 2015/16 to 2017/18 influenza seasons. Euro Surveill. 2019;24.

5. Haas EJ, McLaughlin JM, Khan F, Angulo FJ, Anis E, Lipsitch M, et al. Infections, hospitalisations, and deaths averted via a nationwide vaccination campaign using the Pfizer-BioNTech BNT162b2 mRNA COVID-19 vaccine in Israel: a retrospective surveillance study. Lancet Infect Dis. 2022;22:357–66.

6. COVID-19 open data in Chile. GitHub. https://github.com/MinCiencia/Datos-COVID19. Accessed 16 Apr 2023.
